# Immediate and early loading of hydrothermally treated, hydroxyapatite-coated dental implants: a 7-year prospective randomized clinical study

**DOI:** 10.1186/s40729-021-00299-x

**Published:** 2021-03-10

**Authors:** Afarin Arghami, David Simmons, Jeanne St. Germain, Pooja Maney

**Affiliations:** Department of Periodontics, Louisiana State University Health Sciences Center School of Dentistry, 1100 Florida Avenue, New Orleans, LA 70119 USA

**Keywords:** Dental implants, HA coated, Survival

## Abstract

**Background:**

Existing research on marginal bone stability around hydroxyapatite (HA)-coated implants often lacks adequate long-term follow-up. The purpose of this randomized prospective study was to evaluate the 7-year outcome of patients with immediate and early loaded single-tooth restorations supported by implants with plasma-sprayed, partially HA-coated surfaces. Forty-two patients in need of 50 single implants were treated in in the Postgraduate Periodontics Clinic of Louisiana State University School of Dentistry. Implants were randomly divided into 2 groups: Group A was immediately loaded, and Group B was early loaded. Continuous follow-up with periodic maintenance care and radiographic evaluations was performed. The primary outcome of interest was implant survival, characterized using the Kaplan–Meier method. Secondary study outcome consisted of peri-implant crestal bone level changes. Data on age, sex, bone quality, implant location, length and diameter, and prior augmentation of the site were collected. Multiple regression analyses were conducted to determine whether the independent variables were associated with bone loss.

**Results:**

One implant failed to maintain stability and was removed at 3 weeks. Thirty-four patients (14 males, 20 females with a total of 42 implants) completed the 7-year follow-up visit. Average age of evaluable patients was 52 in Group A and 55 in Group B. No significant difference was observed regarding sex and age distribution between the 2 groups. No significant difference was detected in the distribution of implant locations, types of bone, implant length, implant diameter, and augmentation status of the bone between the 2 groups. After 7 years of functioning for the 42 implants examined, implant survival rate was 100% for Group A and 95.5% for Group B. The results from this study of 50 implants showed that HA-coated Zimmer Tapered Screw-Vent Implants were clinically effective, with an overall cumulative 7-year survival rate of 98.0%. When comparing radiographic bone levels between 2-year and 7-year follow-ups, no significant differences in bone loss were found between Group A and Group B.

**Conclusions:**

After 7 years in function, implants partially coated with plasma-sprayed and hydrothermally treated HA were clinically predictable when restored in occlusion immediately after or 3 weeks after implant placement.

## Background

Endosseous dental implants are currently a widely accepted treatment option for the replacement of missing teeth. The original protocol for treatment with implants proposed by Branemark advocates a waiting period of at least 3 months for osseointegration before loading the implant [1]. However, more recently, immediate or early loading protocols have been successfully implemented [2–5]. Restoring implants immediately or soon after placement is appealing to both patients and clinicians due to the shortened treatment time required.

Esposito et al. defined 3 protocols for implant load timing: immediate implant loading, within 1 week from implant placement; early implant loading, between 1 week and 2 months; and conventional implant loading, after 2 months from implant placement [3]. Although some systematic reviews have shown no convincing evidence of implant failure or bone loss associated with different loading times of implants, other meta-analyses have shown a greater risk for implant failure when compared with conventionally loaded implants [6–8].

The clinical success of immediate loading is dependent on many factors such as bone quality and quantity, implant number and design, implant primary stability, occlusal loading, and clinician’s surgical ability. Among these, implant primary stability is considered the most important [2].

Osstell® (Gothenburg, Sweden) is an electronic instrument designed to measure implant vibrations in response to resonance frequency analysis (RFA). This device measures the resonance frequency of a transductor attached to the implant body [9]. The result of the measurement is the Implant Stability Quotient (ISQ), which corresponds to the hardness of the implant–bone connection [10].

The determinant and most accessible parameter to assess the primary stability is thought to be the implant insertion torque value. However, research has shown strong correlations among primary implant stability, insertion torque (IT) values, and RFA’s proprietary ISQ (Osstell®) [11]. Certain levels of IT and ISQ values have been reported to be suitable indicators for immediate (IT = 35–45 N·cm; ISQ ≥ 70) or early (IT = 30–45 N·cm; ISQ = 40–70) loading of dental implants [12, 13].

Factors such as implant surface characteristics and diameter have also been shown to influence primary stability: rough implant surfaces create more surface area for implant–bone contact [14]. Therefore, implant design and surface also play a role in implant outcomes when loaded immediately or soon after placement.

In the human body, hydroxyapatite (HA) forms 98% of the enamel, 77% of the dentin, 70% of the cementum, and 60–70% of bone by weight [15, 16]. Synthetic HA is a calcium phosphate ceramic that is chemically similar to the HA that forms naturally in the human body. After implantation, it has been reported that calcium phosphate from the implant surface is released into the peri-implant region, which increases the saturation of body fluids and results in the precipitation of a biological apatite layer on the implant surface [17]. Other researchers have reported increased adhesion and proliferation of bone-forming cells at the bone–HA interface in both animal and human models [18–21], which results in accelerated bone formation, maturation, and union between HA-coated implants and the surrounding bone [17].

Longitudinal research is required on the clinical efficacy of plasma-sprayed HA-coated implants with different loading times. The purpose of this prospective study was to evaluate the 7-year outcome of patients with immediate and early loaded single-tooth restorations supported by implants with plasma-sprayed, partially HA-coated surfaces.

## Methods

### Study population

Forty-two patients in need for 50 single implants were treated in in the Postgraduate Periodontics Clinic of Louisiana State University School of Dentistry. The 2-year results of this prospective randomized clinical study were published previously [22]. The current study is a 7-year recall of the same patient population. This protocol was approved by the LSUHSC-NO Institutional Review Board (IRB #7438). The study was conducted in accordance with international standards for health, safety, and good clinical practices, and was adhered to the patient privacy rules of the US Health Insurance Portability and Accountability Act of 1996. This report is structured in alignment with the Consolidated Standards of Reporting Trials (CONSORT) [23].

### Patient selection criteria used

*Inclusion criteria* are males or females; at least 18 years old; healthy enough to undergo routine implant surgery and subsequent dental treatment; partially edentulous requiring single implants in either jaw; adequate bone volume to accommodate implants at least 10-mm long; no active infections; physically, emotionally, and financially able to undergo planned implant procedures; and adequately compliant to meet study requirements and necessary appointments.

*Exclusion criteria* are those with medical need for antibiotic premedication for infective endocarditis; artificial joints or any other medication; uncontrolled hypertension; uncontrolled diabetes; serological human immunodeficiency virus (HIV) positive; history of significant heart, stomach, liver, kidney, blood, immune system or other organ impairment or systemic disease that would prevent undergoing the proposed treatment; smoke cigarettes or other tobacco products; use of investigational drugs during the previous month; unresolved dental conditions likely to require exiting the study for treatment, such as deep cavities, abscesses or moderate to severe periodontal disease; history of radiation therapy to the head and neck; unwilling or inability to sign the informed consent form; failure to demonstrate willingness to return for a required number of visits; and need immediate dental implant placement following tooth extraction.

Dental implants were randomly divided into 2 groups by the study coordinator: in Group A, immediately loaded (implants to be loaded at the day of placement) and in Group B, early loaded (implants to be loaded 3 weeks after placement). Randomization was implemented using assignments in sealed envelopes; these envelopes were opened just prior to each implant surgery, and the patient was assigned to either treatment group based on the information provided in the envelope.

### Surgical procedure

All implants (Tapered Screw-Vent MP-1 HA, Zimmer Dental Inc, Carlsbad, CA) were placed in healed extraction sites with or without prior augmentation by periodontal residents [22]. Implant placement was performed manually using a gauged insertion calibrated torque wrench. The IT value of each implant was recorded in the patient’s chart. Bone density was evaluated by tactile feedback during surgery according to the Lekholm and Zarb scale [24]. RFA (Osstell®) assessment was immediately conducted after implant placement. Two readings were taken with the probe pointing toward the abutment from 2 different directions. An average of the 2 ISQ values was obtained and recorded in the patient’s chart.

### Provisionalization

Patients in Group A received a provisional restoration the day of surgery, loading the implant. Patients in Group B received a healing abutment the day of surgery and returned in 3 weeks. At this time, a provisional restoration was placed, and the implant was loaded. Definitive prosthesis RFA was conducted at 6 months and 12 months of provisionalized loading. After 1 year of provisionalized function, a final impression was made for a computer-aided designed and computer-aided manufactured custom abutment, which was delivered with a definitive, cement-retained crown. Provisional and final restorations were placed by an implant restorative fellow in training.

### Follow-up care

#### Phase I follow-up (6 months to 2 years)

Continuous follow-up with periodic maintenance care was performed every 6 months for 2 years following implant placement. During these periodic follow-up appointments, clinical and radiographic evaluation was performed along with oral hygiene prophylaxis. At every appointment, oral hygiene was reinforced. If patients presented with any problems, they were advised on, and issues were assessed. Periodontal parameters were assessed by clinical evaluation and standardized bitewing digital radiographs (Schick, Sirona Dental Systems, Inc, New York, NY), which were performed using a prefabricated template made for each patient. After this point, patients continued their visits in the periodontics department if any other ongoing treatments existed. Patients with no additional dental treatments at LSUHSC dental clinic were guided to continue receiving oral hygiene prophylaxes elsewhere.

#### Phase II follow-up (at 7 years)

Seven years after the initiation of the study, all participants were contacted and followed up for a 7-year evaluation of the implants. At this follow-up appointment, clinical parameters and restorative complications were assessed by clinical exams and radiographic imaging. The originally made radiographic standardization stents were not reusable anymore due to distortion of the material over the years. Therefore, bitewing radiographs were taken with efforts made to mimic the previous angulations as much as possible for each radiograph image. Clinical and prosthetic evaluations were done at this appointment. All patients were educated with tailored oral hygiene instructions at the end of follow-up visits.

### Evaluation of the bone level changes

Radiographs were taken at 3 different time points: immediately after (baseline) and 2 and 7 years after implant placement. The radiographic images were imported into the ImageJ image processing software. ImageJ is an open source image processing program designed to analyze scientific multidimensional images [25]. Using ImageJ, we measured mesial and distal bone level changes. To calibrate for potential differences in angulation between the radiographs, a known vertical distance which was constant in all subjects (distance from the platform to the first thread of the implant was chosen as reference). All mesial and distal bone levels were corrected using the reference measure (distance from the platform to the first thread of the implant).

### Statistical analysis

Implant survival was summarized through the characterization of failure over time, using the Kaplan–Meier method. Cumulative survival of the implants was estimated at each time of assessment, with corresponding 95% confidence intervals. The secondary study outcome consisted of peri-implant crestal bone level changes (measured from a fixed point on the implant to the area of first bone contact with the implant surface). Peri-implant crestal bone change was measured at the 2- and 7-year follow-up visits. Data on age, sex, bone quality (type 1, 2, 3, or 4) [24], implant location (mandible or maxilla), length and diameter, and prior augmentation of the site were collected. The baseline characteristics of participants were compared between 2 groups using two-sided Student’s *t* test and Fisher’s exact test.

A bivariate analysis was performed to show the distribution of the outcome in different groups. A multivariate regression analysis was conducted to determine whether the independent variables were associated with bone loss at 2 and 7 years. Predictors that, based on the available literature, are potentially associated to bone loss were considered in the full regression model. The predictors included in the full models were (a) type of bone (clinical), (b) location (mandible or maxilla), (c) implant diameter, (d) implant length, (e) load time (immediate or early), (f) augmented or non-augmented bone, (g) ISQ values, (h) participant age (years), and (i) participant sex. A separate regression model was fit for each outcome variable. Participant confidentiality was protected at all stages of the study. Participants’ unique identifiers were used for the analysis.

To estimate the sample size needed for the study, a confidence interval for the sample size of 50 was generated using the expected rate of implant success of 94%. A 95% interval for this rate with a sample size of 50 was 87.4 to 100.0%.

## Results

A total of 42 subjects with 50 dental implants were initially enrolled in this study. One implant failed to maintain stability and was removed at 3 weeks. Thirty-four patients (14 males, 20 females with a total of 42 implants) completed the 7-year follow-up visit (Table 1). One of the male patients had one implant in Group A and 2 implants in Group B. One of the female patients had one implant in Group A and one implant in Group B. Of patients who presented for the 7-year follow-up visit, the average age of patients in Group A was 52 years and in Group B was 55 years (Table 1), ranging between 29 and 73 years. Distribution of the implants based on various clinical and surgical variables is summarized in Table 2.

**Table 1:** Patient demographics by treatment group: 7 year follow-up

	Group A	Group B
*Age (mean years ± SD)	52.28 ± 10.98	55.11 ± 12.18
**Female	11	10
**Male	7	8
Total	18	18

Note: One of the male patients had one implant in Group A and 2 implants in Group B. One of the female patients had one implant in Group A and one implant in Group B. Therefore, the total number of patients reflected in this table (36) does not add up to 34 (actual patient number).

*Independent *t* test

**Fisher’s exact test

**Table 2:** Implant-related variables by treatment group

		No. of implants			
Variable		Total	Group A	Group B	***p*** value
Loading time		42	20	22	
Location	Maxilla	14	5	9	0.275
	Mandible	28	15	13	
Bone augmentation status	Augmented	12	7	5	0.499
	Non-augmented	30	13	17	
Implant length	8 mm	1	1	0	0.76
	10 mm	13	5	8	
	11.5 mm	17	8	9	
	13 mm	11	6	5	
Implant diameter	3.7 mm	5	3	2	0.074
	4.1 mm	14	7	7	
	4.7 mm	17	10	7	
	6.0 mm	6	0	6	
Bone type	Type I	0	0	0	0.717
	Type II	27	12	15	
	Type III	14	7	7	
	Type IV	1	1	0	

*Fisher’s exact test

Of all implants initially assigned to Group A, 20 completed the 7-year follow-up, whereas 22 implants initially assigned to Group B completed the 7-year follow-up. No significant difference was observed regarding the sex (*p* value = 0.5) and age (*p* value = 0.24) distribution between the 2 groups (Table 1). No significant difference was detected in the distribution of implant locations, types of bone in which implants are placed, implant length, implant diameter, and augmentation status of the bone between 2 groups (*p* values > 0.05, Table 2).

At the end of the 7-year follow-up period, 46 patients remained for evaluation (Table 3). The Kaplan–Meier cumulative survival rate of all implants in the study was 98.0% at the 3-week, 6-month, 1-year, 2-year, and 7-year evaluation visits (Table 3). When evaluating survival by cohort, the implant survival was 100% for Group A and 95.5% for Group B (21/22).

**Table 3:** Kaplan–Meier survival analysis: 7 years of follow-up

Follow-up time point	No. of implantsat risk	No. of failed implants	Survival estimate	95% CI survival estimate
3 Weeks	50	1	.9800	(0.8935, 0.9995)
6 Months	49	0	.9800	(0.8935, 0.9995)
1 Year	49	0	.9800	(0.8935, 0.9995)
2 Years	49	0	.9800	(0.8935, 0.9995)
7 Years	46	0	.9800	(0.8935, 0.9995)

The Kaplan–Meier method was used to calculate cumulative implant survival. One implant failed prior to loading at 3 weeks.

### Implant stability measures

**Fig. 1:**
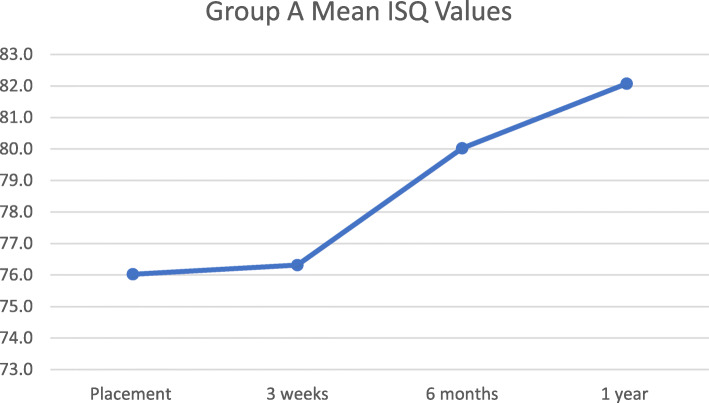
Group A mean ISQ values. This graph depicts change in mean ISQ values for Group A at placement, 3 weeks, 6 months, and 1 year

**Fig. 2:**
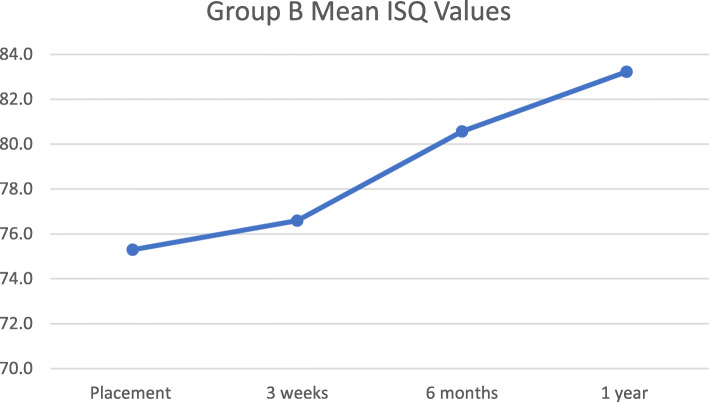
Group B mean ISQ values. This graph depicts change in mean ISQ values for Group B at placement, 3 weeks, 6 months, and 1 year

ISQ values and IT values were measured at the day of implant placement. ISQ values were acquired again at 3 weeks, 6 months, and 1 year after implant placement. Tables 4 and 5 show the average ISQ values at different time points and mean IT values at placement, for Groups A and B. The *t* test showed no significant difference at any time point between the ISQ values of the 2 groups. The *t* test did not show any significant difference between IT values at placement. Mean ISQ values are presented in Figs. 1 and 2, which show an ascending trend over time.

**Table 4:** Mean ISQ values at different time points

	Group A mean ± SD	Group B mean ± SD	***p*** value*
Placement	76.0 ± 4.790	75.3 ± 4.488	0.614
3 Weeks	76.3 ± 3.791	76.6 ± 4.655	0.836
6 Months	80.0 ± 6.034	80.6 ± 6.729	0.784
1 Year	82.1 ± 3.150	83.2 ± 5.149	0.382

*t test

**Table 5:** Mean IT values at placement

	Group A mean ± SD	Group B mean ± SD	***p*** value*
Placement (N·cm)	42.85 ± 9.080	43.81 ± 7.222	0.712

*t test

### Marginal bone loss

Table 6 describes the marginal bone loss at the 2- and 7-year follow-ups compared with baseline, and between 2- and 7-year follow-ups. The cumulative mean radiographic marginal bone loss around the implants after 2 and 7 years in function was 0.414 ± 0.055 mm and 0.498 ± 0.057 mm, respectively, which were not significantly different.

**Table 6:** Marginal bone loss on different follow-up times

Variable		Total	Group A	Group B	***p*** value*
2-Year bone loss (mean ± SD)	Mesial	0.433 ± 0.067	0.428 ± 0.077	0.438 ± 0.110	0.944
	Distal	0.395 ± 0.058	0.395 ± 0.093	0.395 ± 0.075	0.993
	Cumulative	0.414 ± 0.055	0.411 ± 0.076	0.417 ± 0.081	0.962
7-Year bone loss (mean ± SD)	Mesial	0.479 ± 0.072	0.446 ± 0.090	0.510 ± 0.113	0.662
	Distal	0.516 ± 0.053	0.520 ± 0.065	0.512 ± 0.084	0.945
	Cumulative	0.498 ± 0.057	0.483 ± 0.064	0.511 ± 0.094	0.806
2- to 7-year bone loss (mean ± SD)	Mesial	0.046 ± 0.041	0.018 ± 0.072	0.073 ± 0.045	0.513
	Distal	0.121 ± 0.049	0.125 ± 0.088	0.117 ± 0.052	0.935
	Cumulative	0.084 ± 0.039	0.072 ± 0.071	0.095 ± 0.038	0.768

Values are in mm.

*Based on *t* test

Table 7 provides information regarding the test of difference between the mean mesial and distal bone loss in 2 groups. The analysis confirms that there is no significant difference between mesial bone loss and distal bone loss at any point of follow-up time in both groups.

**Table 7:** Comparison between mesial and distal bone loss in the 2 groups

	2-Year bone loss (mean ± SD)			7-Year bone loss (mean ± SD)			2- to 7-year bone loss (mean ± SD)		
	Mesial	Distal	***p*** value	Mesial	Distal	***p*** value	Mesial	Distal	***p*** value*
Group A	0.428 ± 0.077	0.395 ± 0.093	0.669	0.446 ± 0.090	0.520 ± 0.065	0.427	0.018 ± 0.072	0.125 ± 0.088	0.169
Group B	0.438 ± 0.110	0.395 ± 0.075	0.659	0.510 ± 0.113	0.512 ± 0.084	0.974	0.073 ± 0.045	0.117 ± 0.052	0.474

Values are in mm.

*Based on *t* test

The distribution of distal and mesial bone loss at different levels of patient sex, bone augmentation status, implant length, implant diameter, and bone type is shown in Table 8.

**Table 8:** Bone loss at 7 years based on study variables

Variable		Mesial (mean ± SD)	Distal (mean ± SD)	Average (mean ± SD)
Sex	Male	0.500 ± 0.439	0.503 ± 0.371	0.501 ± 0.387
	Female	0.461 ± 0.504	0.528 ± 0.329	0.495 ± 0.363
Bone augmentation status	Augmented	0.305 ± 0.426	0.498 ± 0.383	0.402 ± 0.370
	Non-augmented	0.549 ± 0.474	0.523 ± 0.336	0.536 ± 0.370
Location	Maxilla	0.348 ± 0.537	0.540 ± 0.308	0.444 ± 0.375
	Mandible	0.545 ± 0.426	0.504 ± 0.367	0.524 ± 0.371
Implant length	8 mm	0.430 ± 0.000	0.529 ± 0.000	0.480 ± 0.000
	10 mm	0.463 ± 0.380	0.486 ± 0.363	0.475 ± 0.366
	11.5 mm	0.559 ± 0.540	0.562 ± 0.395	0.561 ± 0.422
	13 mm	0.380 ± 0.489	0.478 ± 0.269	0.429 ± 0.320
Implant diameter	3.7 mm	0.628 ± 0.694	0.702 ± 0.210	0.665 ± 0.447
	4.1 mm	0.370 ± 0.435	0.497 ± 0.340	0.433 ± 0.334
	4.7 mm	0.495 ± 0.451	0.481 ± 0.377	0.488 ± 0.379
	6.0 mm	0.565 ± 0.459	0.503 ± 0.384	0.534 ± 0.413
Bone type	Type I			
	Type II	0.524 ± 0.492	0.522 ± 0.372	0.523 ± 0.400
	Type III	0.358 ± 0.415	0.480 ± 0.298	0.419 ± 0.300
	Type IV	0.959 ± 0.000	0.840 ± 0.000	0.899 ± 0.000
Cumulative		0.480 ± 0.469	0.516 ± 0. 345	0.497 ± 0.370

Values are in mm.

Multivariate regression analysis (Table 9) was applied to analyze the predictors of 2 outcome variables (mean of 2-year bone loss, mean of 7-year bone loss). Regression analysis of the average marginal bone loss indicated that having bone augmentation is correlated with increased bone loss at 2 years (*p* = 0.05). However, such a correlation was not detected at 7 years. Placing an 11.5-mm implant was negatively correlated to bone loss at 2 years (close to significance at *p* = 0.07), indicating that 11.5-mm length might be a predictor for lesser bone loss. However, this was not detected in the 7-year bone loss analysis. Furthermore, having a 4.1-mm diameter implant was significantly correlated to bone loss at 2 years (*p* = 0.04), compared with a smaller diameter (3.7 mm). The same finding was detected in the 7-year bone loss analysis, with at least half a millimeter greater bone loss in 4.1-mm diameter implants (close to significance at *p* = 0.09).

**Table 9:** Multivariate regression analysis on bone loss variables

	Mean bone loss in 2 years (mm)			Mean bone loss in 7 years (mm)		
	Coefficient	SE	***p*** value	Coefficient	SE	***p*** value
**Age**						
< 40 years	Ref.					
40–60 years	0.21	0.27	0.446	0.14	0.31	0.657
> 60 years	0.12	0.28	0.67	0.18	0.31	0.569
**Location**						
Maxilla	Ref.					
Mandible	− 0.03	0.25	0.89	− 0.08	0.28	0.776
**Group**						
A	Ref.					
B	0.17	0.15	0.271	0.05	0.17	0.784
**Gender**						
Female	Ref.					
Male	0.06	0.16	0.73	0.15	0.18	0.415
**Augmentation**	0.36	0.17	0.051	0.20	0.20	0.321
**Mean ISQ**	0.03	0.02	0.174	0.03	0.02	0.214
**Torque**	0.00	0.01	0.757	0.01	0.01	0.609
**Length (mm)**						
8	Ref.					
10	− 0.84	0.50	0.105	− 0.44	0.56	0.441
11.5	− 0.98	0.51	0.068	− 0.60	0.58	0.306
13	− 0.90	0.52	0.098	− 0.53	0.59	0.377
**Diameter (mm)**						
3.7	Ref.					
4.1	0.54	0.25	0.041	0.50	0.28	0.087
4.7	0.35	0.23	0.13	0.26	0.25	0.31
6	0.23	0.27	0.409	0.15	0.30	0.62
**Bone Type**						
2	Ref.					
3	− 0.04	0.17	0.803	0.01	0.19	0.976
4	− 0.31	0.43	0.484	− 0.79	0.49	0.122
***N***	42.00			42.00		

## Discussion

To the authors’ knowledge, this study was the first comparing HA-coated Zimmer Tapered Screw-Vent Implant survival rates and marginal bone stability, when loaded immediately versus when loaded early in 3 weeks. There has been a reluctance to manipulate implants in 3 weeks due to fear of disrupting osseointegration. By using RFA, we were able to dispel this myth. With this implant after 7 years in function, we found no significant loss of stability and with survival of 100% in Group A and 95.5% in Group B. The results from this study of 50 implants showed that HA-coated Zimmer Tapered Screw-Vent Implants were clinically effective, with an overall cumulative 7-year survival rate of 98.0%.This was equal or better than other immediate loading studies [6, 12, 26, 27]. It has been theorized that the layer of apatite that forms of the implant surface during the early stages of osseointegration may (or may not) contain endogenous proteins and serve as a matrix for osteogenic cell attachment and growth on the implant surface [17]. Because the biologic fixation of bone tissue to implant surfaces has been reported in some studies to be faster with a calcium phosphate coating than with uncoated titanium surfaces [18, 19], some clinicians have assumed that the bone healing process around the implant may be enhanced by the formation of the biological apatite layer, which may result in better early stability [17]. All of the implants in the study were stable enough to load at the time of placement. In Group B, all of the implants were stable enough to load at 3 weeks. Stability of all implants increased over the 12-month period.

During the 1990s, the US government conducted a prospective, randomized, multicenter study of HA-coated (*n* = 1725) and uncoated (*n* = 1070) implants placed in participants and monitored for 36–71 months of clinical follow-up [28]. More than 85 dentists in 30 study sites participated, and an independent external review committee composed of experts internationally recognized in their respective fields closely monitored the study [28]. The researchers concluded that HA coating might offer some clinical advantages up to 36 months over uncoated surfaces when placed in poor-quality bone [29], smokers, or when implants were mobile at the time of placement but cautioned that further prospective research was needed to verify these findings. Other researchers in the same study reported that there was no clinically significant difference between in periodontal-type measurements between HA-coated and uncoated dental implants. Nonetheless, the dissolution behaviors of HA coatings with amorphous calcium phosphate phases resulted in relatively isolated reports of possible coating delamination and particle release from the implant surface, which resulted in the clinical failure of the implants [17]. However, a meta-analysis of clinical trials of HA-coated implants found that HA-coated and uncoated implants exhibited no significant differences in survival and success rates [30]. During the mid-1990s, shifting of the calcium phosphate phase was addressed by subjecting HA-coated implants to a hydrothermal treatment that caused their calcium phosphate phase to revert from amorphous to highly crystalline, which significantly helped to resist dissolution [31, 32]. Despite many years of clinical use as a dental implant surface coating, long-term data has remained very limited [17]. A subsequent meta-analysis of clinical trials on HA-coated implants published in 2013 reported that annual failure rates and cumulative survival rates of HA-coated dental implants were comparable to those of non-coated implants [17].

Implant survival rates by group (100% for immediately loaded and 95.5% for early loaded implants) of the present study at the 7-year follow-up period were consistent with earlier outcomes of the same highly crystalline HA-coated surface used on cylindrical rather than threaded implant designs [5, 31].

### Limitations

The current study has limitations. The original sample size was 50 implants, and 8 of these implants were lost to follow-up prior to the study end, decreasing the evaluable sample size and with unknown outcomes for the censored implants. Additionally, the study was conducted at a single center, so operator (surgeon) influence, either positive or negative, could not be considered as a covariate in the analyses. Lastly, this study did not include smokers or those with significant comorbidities, so it is unknown if the implant performance that was noted in this study is generalizable to a larger and heterogenous population.

## Conclusion

After 7 years in function, implants partially coated with plasma-sprayed and hydrothermally treated HA were favorable when restored in occlusion immediately after or after 3 weeks of implant placement. Overall, within the limitations of our study, the outcomes of this particular implant system were favorable with clinically predictable results at 7 years for both immediate and early loaded implants.

## Data Availability

The datasets generated and/or analyzed during the current study are not publicly available as they were obtained during clinical practice; however, the data are available from the corresponding author on reasonable request.

## Abbreviations

CONSORT: Consolidated Standards of Reporting Trials
HA: Hydroxyapatite
IRB: Institutional Review Board
ISQ: Implant stability quotient
IT: Insertion torque
LSUHSC-NO: Louisiana State University Health Science Center—New Orleans
RFA: Resonance frequency analysis
